# RET PLCγ Phosphotyrosine Binding Domain Regulates Ca^2+^ Signaling and Neocortical Neuronal Migration

**DOI:** 10.1371/journal.pone.0031258

**Published:** 2012-02-15

**Authors:** T. Kalle Lundgren, Katsutoshi Nakahata, Nicolas Fritz, Paola Rebellato, Songbai Zhang, Per Uhlén

**Affiliations:** 1 Department of Medical Biochemistry and Biophysics, Karolinska Institutet, Stockholm, Sweden; 2 Department of Reconstructive Plastic Surgery, Karolinska University Hospital, Stockholm, Sweden; Seattle Children's Research Institute, United States of America

## Abstract

The receptor tyrosine kinase RET plays an essential role during embryogenesis in regulating cell proliferation, differentiation, and migration. Upon glial cell line-derived neurotrophic factor (GDNF) stimulation, RET can trigger multiple intracellular signaling pathways that in concert activate various downstream effectors. Here we report that the RET receptor induces calcium (Ca^2+^) signaling and regulates neocortical neuronal progenitor migration through the Phospholipase-C gamma (PLCγ) binding domain Tyr1015. This signaling cascade releases Ca^2+^ from the endoplasmic reticulum through the inositol 1,4,5-trisphosphate receptor and stimulates phosphorylation of ERK1/2 and CaMKII. A point mutation at Tyr1015 on RET or small interfering RNA gene silencing of PLCγ block the GDNF-induced signaling cascade. Delivery of the RET mutation to neuronal progenitors in the embryonic ventricular zone using *in utero* electroporation reveal that Tyr1015 is necessary for GDNF-stimulated migration of neurons to the cortical plate. These findings demonstrate a novel RET mediated signaling pathway that elevates cytosolic Ca^2+^ and modulates neuronal migration in the developing neocortex through the PLCγ binding domain Tyr1015.

## Introduction

RET (REarranged during Transfection) was initially identified as an oncogene [1], but several additional important functions during development and disease have since been discovered [2], [3], [4]. The RET gene, on human chromosome 10q11.2, encodes a receptor tyrosine kinase that is activated by the glial cell line-derived neurotrophic factor (GDNF) family of ligands in conjunction with ligand-specific co-receptors of the GDNF-family receptor-α (GFRα) [5], [6]. GDNF/GFRα-activation of RET results in transphosphorylation of tyrosine residues in its intracellular kinase domain that triggers multiple intracellular signaling pathways that in concert regulate cell proliferation, migration, differentiation, survival, neurite outgrowth, and synaptic plasticity [2]. Loss-of-function mutations in RET cause Hirschsprung's disease, a developmental disorder of the enteric nervous system [7], whereas gain-of-function mutations cause multiple endocrine neoplasia type 2a or b (MEN2a/b), a dominantly inherited cancer syndrome [8]. RET mediated signaling in the nervous system has for the most part been studied in cell lineages derived from the neural crest [9]. However, since both GDNF, GFRα1 and RET are expressed in the embryonic neocortex [10], there is a growing interest in understanding the role of RET and its ligands in the central nervous system [11], [12], [13].

The intracellular domain of the RET protein has several tyrosine residues that become auto-phosphorylated upon ligand interaction and mediate activation of various downstream signaling targets, including the mitogen-activated protein kinase (MAPK) [3], [14] and the calcium/calmodulin-dependent protein kinase II (CaMKII) [15]. Mutating RET tyrosine residue 1062 (Tyr1062) gives a phenotype that largely resembles RET deletion mutants [16], [17]. Phosphorylated Tyr1062 tethers transduction effectors (including SHC, FRS2 and IRS1 family proteins [2]) to activate several signaling pathways including the Phosphatidylinositol 3-kinase (PI3K)/Akt and Ras/MAPK cascades [7]. A different RET tyrosine residue, Tyr1015, stimulates the phospholipase C γ (PLCγ) pathway [18]. Mice bearing Tyr1015 point mutation resulting in disrupted PLCγ activation show abnormal kidney development and death at 1 month of age [19]. While these findings have expanded our understanding of RET Tyr1015, little is known about downstream signaling pathways activated by RET-phosphorylated PLCγ. One potential signaling pathway that is modulated by PLCγ is cytosolic calcium (Ca^2+^) signaling.

The Ca^2+^ ion serves as a universal cytosolic messenger to control a diverse range of cellular processes in both disease and development [20], [21]. Transporters of Ca^2+^ handle the temporal and spatial distribution of cytosolic Ca^2+^ by regulating influx and efflux from the extracellular milieu or release from the endoplasmic reticulum (ER) stores [22], [23]. Release of Ca^2+^ from ER mainly occurs through the inositol 1,4,5-trisphosphate (InsP_3_) receptor (InsP_3_R). The InsP_3_R is activated by Ca^2+^ itself or by InsP_3_ that is produced when PLC cleaves phosphatidylinositol 4,5-bisphosphate. An elevated cytosolic Ca^2+^ concentration triggers various downstream effectors such as MAPK and CaMKII, which subsequently modulate cellular processes including neuronal migration, axon and dendrite development and regeneration, and synaptic plasticity [23], [24], [25].

We here demonstrate that RET receptor activation by GDNF stimulates cytosolic Ca^2+^ signaling through a PLCγ phosphotyrosine binding site at Tyr1015. This GDNF/RET/PLCγ/InsP_3_R signaling cascade triggers release of Ca^2+^ from internal ER stores that subsequently phosphorylates p42/44 of MAPK (ERK1/2) and CaMKII. Additionally, we report that RET is present in the neocortex of the developing brain and that overexpressing a RET Tyr1015 point mutation perturbs GDNF-stimulated migration of neocortical neuronal progenitor cells.

## Results

### Calcium Signaling

**Figure 1:**
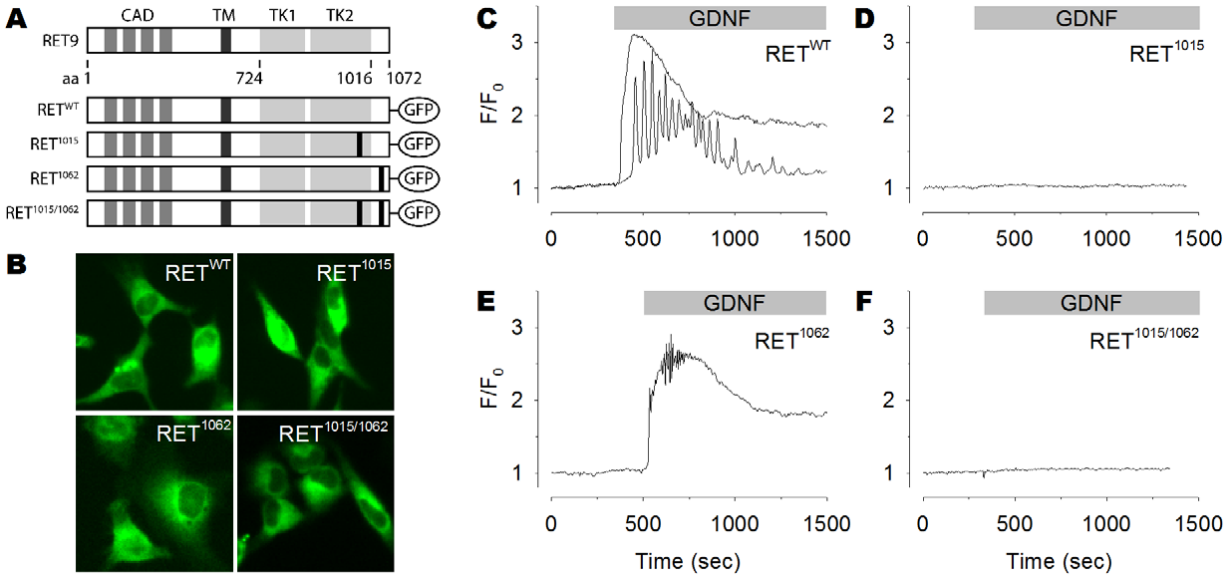
GDNF induces cytosolic Ca^2+^ signaling through Tyr1015 of RET. (**A**) Schematic representation of GFP-tagged RET constructs. (**B**) Constructs expressed in HeLa cells. (**C–F**) Representative single-cell Ca^2+^ recordings of GFP positive HeLa cells loaded with Fura-2/AM and subsequently treated with GDNF (100 ng/ml). (**C**) Cells expressing the RET^WT^ construct responded to GDNF with either Ca^2+^ transient (top trace) or Ca^2+^ oscillations (bottom trace). Cells expressing RET^1015^ (**D**) or RET^1015/1062^ (**F**) failed to trigger a Ca^2+^ response following GDNF exposure. Cells expressing RET^1062^ (**E**) responded to GDNF in a similar manner as cells expressing RET^WT^.

Single-cell live Ca^2+^ imaging in HeLa cells was used to determine whether the RET receptor was involved in cytosolic Ca^2+^ signaling. Cells were transfected with green fluorescent protein (GFP)-tagged wild-type RET (RET^WT^) 24 h prior to loading with the Ca^2+^-sensitive dye Fura-2/AM (Figure 1A and B). The cytosolic Ca^2+^ concentration was exclusively examined in GFP positive cells. Treatment with GDNF and GFRα1 in RET^WT^ expressing cells resulted in a rapid cytosolic Ca^2+^ increase in 58% of the cells whereas an oscillatory Ca^2+^ response was observed in 25% of the cells (Table 1 and Figure 1C).

**Table 1:** Characteristics of Ca^2+^ responses induced by GDNF in cells expressing various RET constructs.

		non-responding^a^	transient^b^	oscillation^c^	
		% (*n*)	% (*n*)	% (*n*)	[*n*/*N*]^d^
GDNF/GFRα1	GFP	99.0 (312)	0.6 (2)	0.3 (1)	[315/5]
	RET^WT^	17.0 (66)	58.1 (226)	24.9 (97)	[389/6]
	RET^1015^	99.1 (338)	0.6 (2)	0.3 (1)	[341/6]
	RET^1062^	9.2 (33)	66.0 (237)	24.8 (89)	[359/5]
	RET^1015/1062^	100.0 (421)	0 (0)	0 (0)	[421/5]

a Non-responding cells have no Ca^2+^ increase exceeding 1.25 of the baseline.

b Transient responding cells have one Ca^2+^ peak exceeding 1.25 of the baseline.

c Oscillatory responding cells have at least three Ca^2+^ peaks exceeding 1.25 of the baseline.

d [number of cells/number of experiments].

Equivalent GFP-tagged RET constructs, bearing point mutations at tyrosine residues at positions 1062 (RET^1062^), 1015 (RET^1015^) or both (RET^1062/1015^), were used to identify a tyrosine residue that mediated the Ca^2+^ response (Figure 1A and Figure S1A). The RET^1015^ mutation abolished all cytosolic Ca^2+^ elevation (Table 1 and Figure 1D), whereas the RET^1062^ mutation had no significant effect on the Ca^2+^ response triggered by GDNF (Table 1 and Figure 1E). Cells expressing the double point mutation RET^1015/1062^ also failed to evoke a Ca^2+^ increase (Table 1 and Figure 1F). RET receptor-activation by GDNF therefore triggers cellular Ca^2+^ responses by a molecular mechanism involving Tyr1015, but not Tyr1062.

### Signaling Pathway

**Figure 2:**
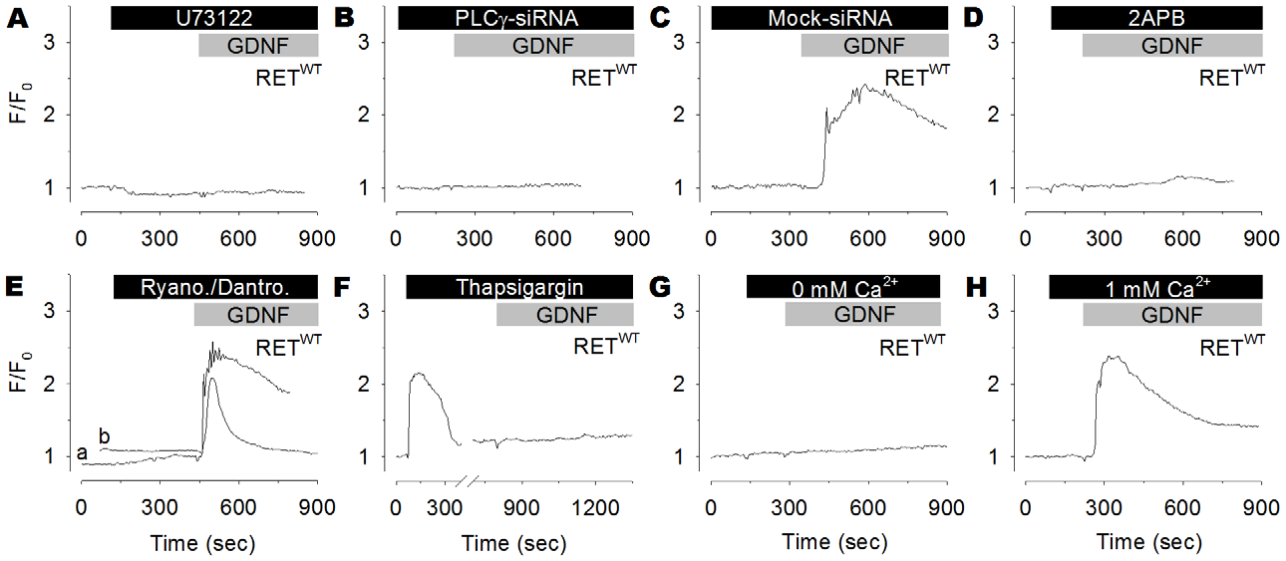
A RET/PLCγ/InsP_3_R-cascade stimulates GDNF-induced Ca^2+^ release. (**A–H**) Representative single-cell Ca^2+^ recordings of GFP positive RET^WT^ expressing cells loaded with Fura-2/AM and preincubated with inhibitors as indicated, following treatment with GDNF (100 ng/ml). Inhibiting PLC with U73122 (5 µM) (**A**) or knocking down PLCγ with siRNA (**B**) blocked the cytosolic Ca^2+^ response induced by GDNF. Cells transfected with the Mock-siRNA retain the Ca^2+^ response (**C**). Inhibiting InsP_3_R with 2-APB (5 µM) abolished the Ca^2+^ response induced by GDNF (**D**), while inhibiting RyR with ryanodine (a, 20 µM) or dantrolene (b, 10 µM) had no effect (**E**). Depleting intracellular Ca^2+^ stores with the SERCA Ca^2+^-ATPase inhibitor Thapsigargin (1 µM) blocked the Ca^2+^ response (**F**). Zero extracellular Ca^2+^ eliminated the GDNF-induced Ca^2+^ response (**G**), whereas a low extracellular concentration of Ca^2+^ (1 mM) produced a normal Ca^2+^ response (**H**).

The mechanism by which GDNF stimulated Ca^2+^ signaling was determined by using single cell Ca^2+^ recordings while blocking various known Ca^2+^-regulators with small molecule inhibitors or small interfering RNA (siRNA). Tyr1015 is known to bind PLCγ to the RET receptor, suggesting that PLC played a role in this signaling pathway. The effect of the PLC-inhibitor U73122 was therefore examined. GDNF failed to elevate cytosolic Ca^2+^ in RET^WT^ expressing cells that were pre-treated with U73122 (Table 2 and Figure 2A). A siRNA against the PLCγ mRNA was used as an alternative method to ablate the PLCγ function. The PLCγ-siRNA drastically reduced PLCγ (Figure S1B) and blocked the GDNF-induced Ca^2+^ increase in cells expressing RET^WT^ (Table 2 and Figure 2B). The mock-siRNA failed to abolish the Ca^2+^ response (Figure 2C).

**Table 2:** Characteristics of Ca^2+^ responses triggered by GDNF in RET^WT^ cells treated with various inhibitors.

		non-responding^a^	transient^b^	oscillation^c^	
		% (*n*)	% (*n*)	% (*n*)	[*n*/*N*]^d^
RET^WT^+GDNF/GFRα1	vehicle	8.5 (11)	77.5 (100)	14.0 (18)	[129/3]
	U73122	98.6 (139)	1.4 (2)	0 (0)	[141/3]
	PLCγ-siRNA	96.9 (62)	3.1 (2)	0 (0)	[64/6]
	2-APB	54.9 (67)	36.9 (45)	8.2 (10)	[122/3]
	Ryanodine	25.0 (21)	66.7 (56)	8.3 (7)	[84/3]
	Thapsigargin	96.8 (150)	2.7 (4)	0.6 (1)	[155/3]
	Ca^2+^ [0 mM]^e^	98.9 (176)	1.1 (2)	0 (0)	[178/3]
	Ca^2+^ [1 mM]^e^	22.6 (26)	64.3 (74)	13.0 (15)	[115/3]

a Non-responding cells have no Ca^2+^ increase exceeding 1.25 of the baseline.

b Transient responding cells have one Ca^2+^ peak exceeding 1.25 of the baseline.

c Oscillatory responding cells have at least three Ca^2+^ peaks exceeding 1.25 of the baseline.

d [number of cells/number of experiments].

e [concentration of extracellular Ca^2+^].

Two key proteins involved in Ca^2+^ release from the ER are the InsP_3_R and the Ryanodine receptor (RyR). Exposing RET^WT^ expressing cells to the InsP_3_R inhibitor 2-aminoethoxydiphenyl borate (2-APB) blocked the cytosolic Ca^2+^ increase triggered by GDNF (Table 2 and Figure 2D). In contrast, preincubating cells with Ryanodine, which prevents Ca^2+^ release through RyR, produced no significant change in the Ca^2+^ response (Table 2 and Figure 2Ea). Another RyR blocker Dantrolene, also failed to inhibit the GDNF-induced Ca^2+^ response (Figure 2Eb).

Blocking the sarco/endoplasmic reticulum Ca^2+^-ATPase (SERCA) pump depletes the ER of Ca^2+^, so that cytosolic Ca^2+^ can no longer be elevated from ER Ca^2+^ stores. Pre-treatment of RET^WT^ expressing cells with the SERCA pump inhibitor Thapsigargin triggered a typical cytosolic transient Ca^2+^ increase as the ER stores depleted. Subsequent GDNF exposure failed to elevate free cytosolic Ca^2+^ (Table 2 and Figure 2F). GDNF-induced cytosolic Ca^2+^ signaling therefore appeared to come from the ER.

The contribution of extracellular Ca^2+^ was also investigated by recordings in Ca^2+^ free medium. The GDNF-induced cytosolic Ca^2+^ response was abolished in RET^WT^ expressing cells when extracellular Ca^2+^ was removed (Table 2 and Figure 2G). This result initially suggested that the GDNF-triggered Ca^2+^ response may also derive Ca^2+^ from extracellular sources. However, the RET receptor has four extracellular cadherin-like domains that contain Ca^2+^-binding sites. Thus, extracellular Ca^2+^ ions are required for correct structural alignment of the RET receptor [26], [27]. It was hence possible that the elimination of Ca^2+^ from the medium caused a structural defect in RET. Experiments were therefore repeated with lower concentrations of Ca^2+^ in the medium. A GDNF-induced Ca^2+^ response was observed with as little as 1 mM of extracellular Ca^2+^ (Table 2 and Figure 2H). Taken together, these data suggest that the GDNF-induced Ca^2+^ response comes from ER Ca^2+^ stores rather than from the extracellular milieu.

### Downstream Effectors

Two proteins, MAPK and CaMKII, are typically phosphorylated when free cytosolic Ca^2+^ levels increase. Experiments were undertaken to determine if the cytosolic Ca^2+^ signal induced by the GDNF/RET/PLCγ/InsP_3_R-mediated cascade of our model system could influence these two downstream effectors.

**Figure 3:**
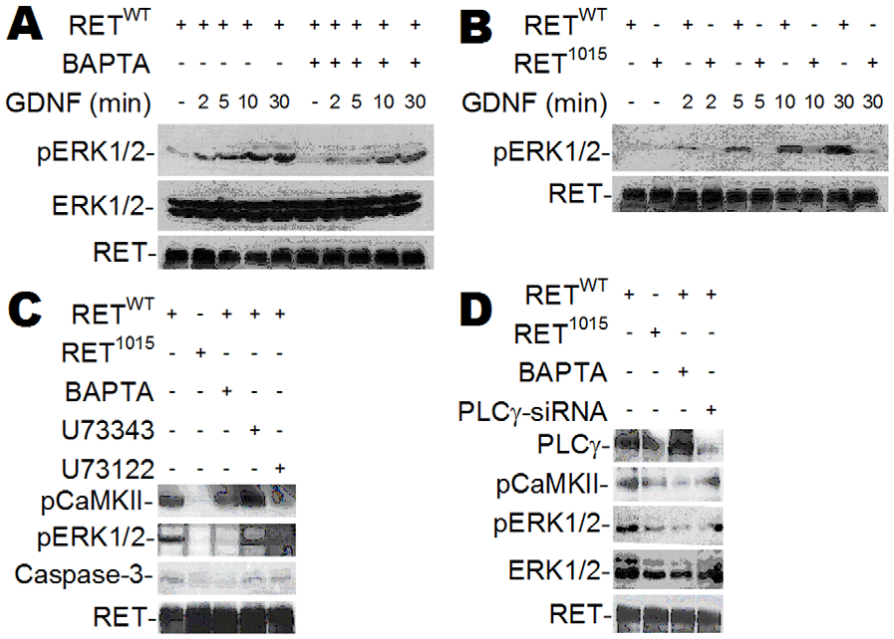
GDNF-induced Ca^2+^ signaling phosphorylates ERK1/2 and CaMKII. (**A**–**D**) Western blot of HeLa cells transfected with RET^WT^ or RET^1015^ treated with GDNF (100 ng/ml). GDNF triggers time dependent phosphorylation of ERK1/2 (pERK1/2) in RET^WT^ cells that is suppressed by BAPTA (10 µM) (**A**). Less pERK1/2 is observed in cells transfected with RET^1015^ than RET^WT^ (**B**). GDNF-induced phosphorylation of CaMKII (pCaMKII) or pERK1/2 is suppressed when blocking PLC with U73122 (5 µM) (**C**) or knocking down PLCγ with siRNA (PLCγ-siRNA) (**D**). Treating RET^WT^ cells with the U73122 analogue U73343 (5 µM) had no effect on GDNF-activated pCaMKII or pERK1/2 (**C**). Increased Caspase-3 cleavage was not detected in cells treated with the inhibitors BAPTA or U73122 (**C**).

Phosphorylation of ERK1/2 was followed on Western blots. Cells transfected with RET^WT^ and exposed to GDNF for 2 to 30 min showed time-dependent ERK1/2 phosphorylation (Figure 3A). Less ERK1/2 phosphorylation was observed in the presence of BAPTA, which sequesters free cytosolic Ca^2+^ (Figure 3A). Indeed BAPTA completely abolished the elevation of cytosolic Ca^2+^ induced by GDNF (Figure S2). Parallel time course experiments showed that the mutation of Tyr1015 severely reduced or abolished the ability of RET to induce ERK1/2 phosphorylation (Figure 3B).

CaMKII was also phosphorylated when cells transfected with RET^WT^ were exposed to GDNF (Figure 3C). In contrast, phosphorylation was not observed at all in cells transfected with the RET^1015^ mutation (Figure 3C). The GDNF-evoked CaMKII phosphorylation was slightly attenuated by sequestering free cytosolic Ca^2+^ with BAPTA or by inhibiting PLC with U73122 (Figure 3C). The U73122 analogue, U73343, which does not inhibit PLC, did not block phosphorylation (Figure 3C). This set of experiments suggested that RET-dependent phosphorylation of ERK1/2 and CaMKII was, at least in part, induced by elevated levels of Ca^2+^.

PLCγ-siRNA was thereafter applied to further investigate PLC-mediated ERK1/2 and CaMKII phosphorylation. Knocking down PLCγ with siRNA suppressed phosphorylation of ERK1/2 and CaMKII in cells transfected with RET^WT^ and exposed to GDNF (Figure 3D).

In summary, these results indicate that GDNF/RET-induced Ca^2+^ signaling phosphorylates ERK1/2 and CaMKII by a mechanism that absolutely depends on a Tyr at amino acid 1015 of the RET receptor.

### Cell Motility

**Figure 4:**
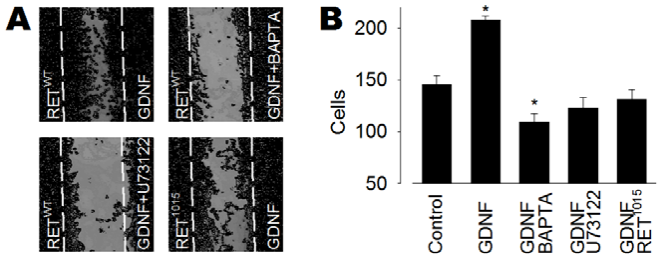
GDNF-induced Ca^2**+**^ signaling stimulates cell motility *in vitro*. (**A**) Cell motility assay in HeLa cells transfected with RET^WT^ or RET^1015^ and treated with GDNF (100 ng/ml) for 6–8 h. (**B**) Cell motility was significantly higher in RET^WT^ transfected cells treated with GDNF, as compared to control cells without GDNF. Buffering cytosolic Ca^2+^ with BAPTA (10 µM) or inhibiting PLC with U73122 (5 µM) abolished the cell motility. GDNF failed to stimulate cell motility in cell transfected with RET^1015^. Bars represent the average number of cells in the scratch. * *P*<0.05 versus control.

Since GDNF/RET has been reported to regulate cell migration [9], experiments were performed to examine whether GDNF/RET-induced Ca^2+^ signaling influenced cell motility. A wound healing assay was used, in which HeLa cells were grown to confluence and a caliper-measured scratch was made in the adherent cell layer (Figure 4A). In the absence of GDNF, RET^WT^ transfected cells failed to move over the scratched area in the next 6–8 h (Figure 4B). However, when GDNF was included in the medium the scratch was populated by cells (Figure 4A and B). Treating the RET^WT^ transfected cells with BAPTA or U73122 significantly inhibited the observed effect (Figure 4A and B). BAPTA and U73122 did not stimulate early apoptosis, as no significant level of cleaved Caspase-3 was detected by Western blot (Figure 3C). Cells transfected with the RET^1015^ mutant showed significantly fewer cells in the scratched area after treatment with GNDF than cells transfected with RET^WT^ (Figure 4A and B). RET^1015^ did not induce significant Caspase-3 cleavage (Figure 3C), which suggested that the decreased number of cells in the scratched area was likely to be an effect of reduced cell motility caused by the Tyr1015 mutation.

**Figure 5:**
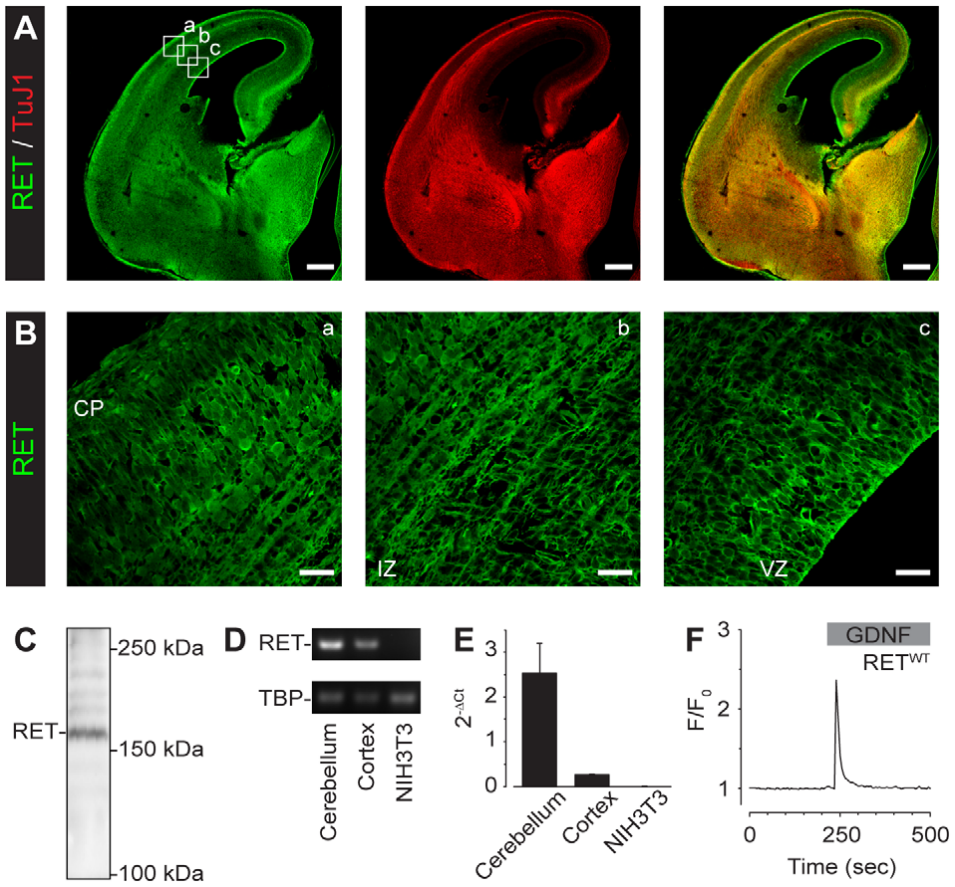
Endogenous RET is expressed in the embryonic neocortex. Immunohistochemistry of an E14.5 mouse forebrain coronal slice (**A**, Scale bars, 250 µm) and cortical plate (CP), intermediate zone (IZ) and ventricular zone (VZ) regions (**B**, Scale bars, 25 µm) for RET and TuJ1. Western blot (**C**), reverse transcription PCR (35 cycles) (**D**) and real-time PCR (**E**) analysis for RET in cortical tissue. Cerebellar tissue and NIH3T3 cells were used as controls. TATA-box binding protein (TBP) was the house keeping gene. (**F**) Representative single-cell Ca^2+^ recording of a RET^WT^ expressing primary cortical neuron loaded with Fura-2/AM and subsequently treated with GDNF (100 ng/ml).

**Figure 6:**
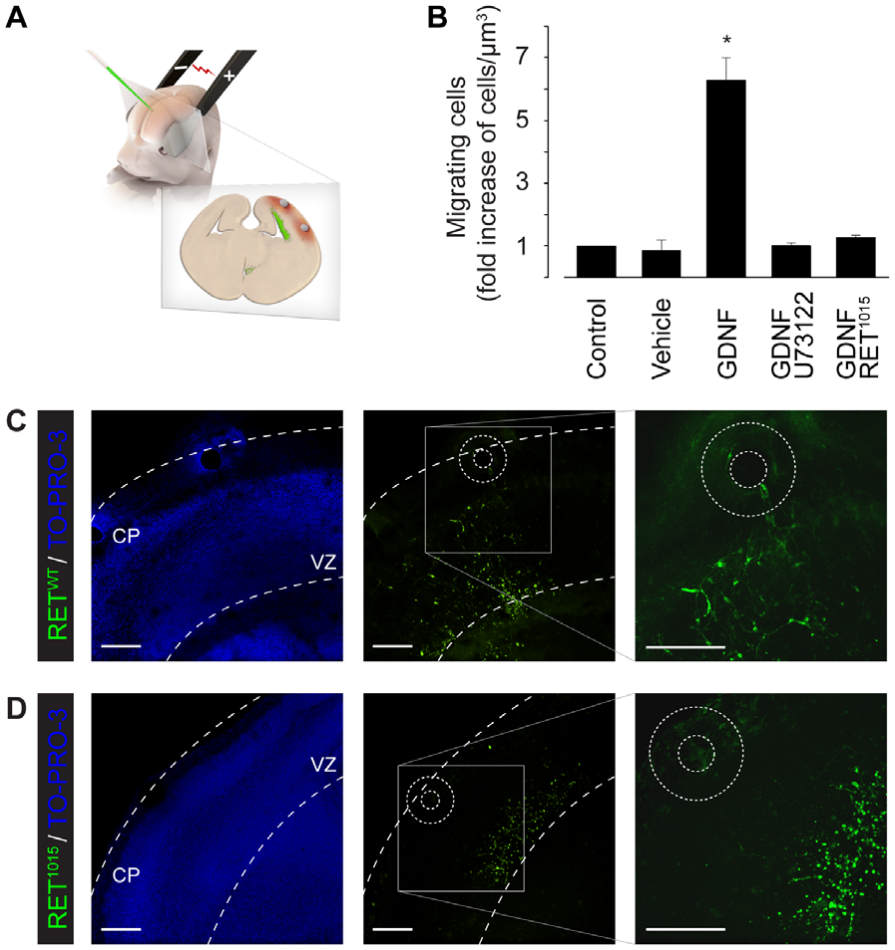
RET Tyr1015 mediates GDNF-stimulated migration *in vivo*. (**A**) Cartoon illustrating mouse embryo electroporation and GDNF-bead stimulated migration. (**B**–**D**) Migration of cortical progenitors in organotypic brain slices from embryos electroporated with RET^WT^ (**C**) or RET^1015^ (**D**) treated without beads (Control) or with beads (indicated with circles) soaked in PBS (Vehicle) or GDNF (500 ng/ml) placed in the cortical plate (CP). GFP positive RET^WT^ expressing progenitors (green) stimulated with GDNF beads (**B**, **C**) show significantly enhanced migration from the ventricular zone (VZ) towards the CP, as compared to Control, Vehicle, or inhibition of PLC with U73122 (5 µM). In RET^1015^ expressing progenitors GDNF beads failed to stimulate migration (**B**, **D**). Scale bars, 100 µm.

To explore the biological relevance of GDNF/RET-induced Ca^2+^ signaling, we next performed experiments using an *in vivo* model of neocortical migration. Immunohistochemistry on mouse E14.5 brain coronal slices revealed a homogenous RET expression in the embryonic neocortex (Figure 5A and B). The neural stem cells of the ventricular zone and more differentiated cells in the intermediate zone (IZ) and cortical plate were all expressing RET (Figure 5B and Figure S3). Western blotting (Figure 5C) and reverse transcription-PCR (Figure 5D), in accordance with the results obtained by Ibáñez and co-workers [10], showed that neural cells of the cortex were expressing endogenous RET. Cerebellum, which is known to express high levels of RET, was used as positive control [28], whereas NIH3T3 cells was used as negative control [29]. A quantitative measure of mRNA levels using real-time PCR revealed a weaker, but significant, expression of RET in the embryonic cortex (Figure 5E). Primary cultures of cerebral cortical neurons at E14.5 loaded with Fura-2/AM responded to GDNF (100 ng/ml) with a rapid Ca^2+^ response in 8.6% (*n* = 151) of the cells. Expressing RET^WT^ in primary cortical neurons produced a rapid Ca^2+^ response in 12.9% (*n* = 31) of the cells (Figure 5F) whereas none of the cells expressing RET^1015^ responded to GDNF (*n* = 34). *Ex utero* electroporation was then performed to determine whether the Tyr1015 of RET played a role for neocortical neuronal migration. RET^WT^ or RET^1015^ constructs were injected into the lateral ventricles of E14.5 embryonic forebrains and electroporated *ex utero* (Figure 6A). Organotypic slice cultures were thereafter prepared from the electroporated embryos and beads soaked in GDNF (500 ng/ml) were placed on top of the cortical plate (CP) for 48–72 h (Figure 6A). Confocal z-stack images of electroporated regions were then recorded and GFP-positive neuronal progenitor cells in the ventricular zone (VZ) of the neocortex were analyzed for migration. RET^WT^ expressing cells showed a significant 6.3±0.7-fold (*n* = 5) increase in migration towards the GDNF-beads in the CP, as compared to control regions and vehicle (Figure 6B and C). Blocking PLC with U73122 significantly inhibited the GDNF/RET-stimulated migratory movement (1.0±0.1-fold increase, *n* = 6) of RET^WT^ expressing cells (Figure 6B). Treating slices with U73343, a U73122 analogue that does not inhibit PLC, did not inhibit the observed migration (5.0±0.4-fold increase, *n* = 6). These results indicated that PLC-dependent GDNF/RET-signaling played a role in the migratory movement of neuronal progenitor cells overexpressing RET in the VZ.

The RET^1015^ construct was thereafter delivered into the embryonic VZ progenitor cells to further test the influence of RET Tyr1015 on neuronal migration. GDNF-beads in the CP failed to stimulate migration (0.3±0.1-fold increase, *n* = 4) of neocortical neuronal progenitors expressing the RET^1015^ construct (Figure 6B and D).

In conclusion, our results demonstrate that RET is expressed in the embryonic neocortex and that GDNF-stimulated neocortical progenitor migration in the developing brain is modulated by Tyr1015 of the RET receptor.

## Discussion

In the present study we show that GDNF evokes cytosolic Ca^2+^ signaling by releasing Ca^2+^ from ER stores. The release is dependent on RET, PLCγ, and InsP_3_R and modulates ERK1/2 and CaMKII phosphorylation. The signaling cascade is mediated by a single residue of RET since a point mutation of Tyr1015 fails to nitiate the signaling event. Delivery of the RET Tyr1015 mutant DNA to HeLa cells or neuronal progenitors in the VZ of mouse embryos impairs GDNF-stimulated cell motility *in vitro* as well as *in vivo*.

The clinical relevance of RET was established when it was shown that germline mutations of the RET gene were responsible for two inherited human disorders, those being Hirschsprung's disease and MEN2a/b [3], [8]. Hirschsprung's disease is a complex developmental genetic disorder characterized by the absence of enteric ganglia in the intestinal tract, whereas MEN2a/b is a cancer syndrome that affects neuroendocrine organs. Various mutations in the RET gene have been identified and correlated with these two disorders [2]. Interestingly, the disease phenotypes of Hirschsprung's disease and MEN2a/b show partial resemblance with previously reported Ca^2+^ signaling-dependent disorders [30], [31], [32], [33]. Thus, RET regulated Ca^2+^ signaling may be involved in Hirschsprung's disease and MEN2a/b. In the case of RET as an oncogene the results presented herein might be of clinical benefit as cytosolic Ca^2+^ signaling is implicated in general cancer growth as well as in thyroid cancers of the MEN2b type [30], [34], [35]. Tumor cell proliferation has recently been reported in papillary thyroid carcinoma through a signaling pathway where RET, MAPK, and CaMKII contributes [15]. However, this study is the first report of RET-induced Ca^2+^ signaling dependent on a specific phosphotyrosine and will contribute to the overall understanding of RET-regulated cell mechanisms in human diseases.

Tyr1062 of RET mediates most of the well characterized interaction with different adaptor proteins [36], [37]. However, several phosphorylation sites exist and may work independently or in concert to activate certain cellular processes. For example, a synchronized activation of RET Tyr905, Tyr1015, and Tyr1062 has been detected in embryonic mouse dorsal root ganglia [36]. Phosphorylation of RET Tyr1015 activates the PLCγ pathway [18] and is important in kidney development because mutation of this residue abolishes the otherwise competent rescue of Tyr1062 mutations by Tyr1096 in the long isoform of RET [19]. Mutation of RET Tyr697, a putative protein kinase A (PKA) phosphorylation site, causes migration defects in enteric neural crest cells [38]. This and other observations [39] show a link between cyclic AMP (cAMP) and RET mediated cell signaling. cAMP levels also affect cytosolic Ca^2+^ signaling influences neuronal survival, regeneration, and growth cone remodeling [40], [41], [42]. Our results demonstrate that RET Tyr1015 mediates a GDNF-triggered increase in cytosolic Ca^2+^ and modulates neuronal progenitor migration in the embryonic neocortex. The demonstration of RET expression and function in the developing brain raise the possibility that the RET receptor plays an important role in the embryonic cortex.

GDNF/RET has previously been reported to modulate differentiation and migration through multiple cell signaling pathways. For example, migration of enteric nervous system progenitor cells and cortical GABAergic neurons has been linked to the pathways of Ras/ERK and PI3K/Akt [10], [12], [43], respectively. The GDNF-stimulated tangential migration of GABAergic neurons was dependent of GFRα1 but not of RET [10]. Moreover, in a recent study, mice with a homozygous deletion of the kinesin superfamily protein 26A (KIF26A^−/−^) were shown to have perturbed enteric neuronal development as a result of hypersensitivity to RET signaling [44]. Also Akt/ERK signaling played an essential role for GDNF/RET-dependent enteric neuronal development in the KIF26A^−/−^ mice. Neurite outgrowth in human neuroblastoma cells stimulated by RET was shown to be regulated through downstream activation of Ras/ERK [14]. A recent study suggests that ERK-dependent GDNF/RET-induced neurite outgrowth is suppressed by the RET-binding protein Rap1GAP [45]. In somatotrophs, the pituitary cells secreting growth hormones during infancy and puberty, activation of protein kinase C (PKC) and cAMP response element-binding (CREB) transcription factor are regulated through RET-mediated signaling pathway [46]. Mice lacking the RET receptor display early differentiation defects of the dorsal root ganglia somatosensory neurons [47]. Interestingly, all the proteins Akt, CREB, ERK, PI3K, PKC, and Ras are known to be partially regulated by Ca^2+^ signaling [22]. A connection between GDNF/RET and Robo2/Slit2, yet another signaling pathway known to be regulated by Ca^2+^ signaling [48], in promoting ureteric bud outgrowth has been reported [49]. Nonetheless, how GDNF/RET triggers Slit2/Robo2 signaling is not clear but might be attributed to Ca^2+^ signaling. Such a Ca^2+^ signaling link between RET and Slit2/Robo2 could, at least in part, be involved in the GDNF-directed neuronal migration in neocortex of the developing brain.

Our results demonstrate a novel RET signaling pathway where GDNF stimulates cytosolic Ca^2+^ signaling through the PLCγ phosphotyrosine binding site Tyr1015 of RET. This GDNF/RET/PLCγ/InsP_3_R signaling cascade elevates the cytosolic Ca^2+^ concentration by releasing Ca^2+^ from internal ER stores. The cytosolic Ca^2+^ response mediated through Tyr1015 of RET subsequently phosphorylates the downstream effectors ERK1/2 and CaMKII. Our data also show that RET is homogenously expressed in the cortex of the developing brain. Mutating Tyr1015 and delivering the DNA to neuronal progenitors in the VZ of mouse embryos impairs GDNF-stimulated migration in the developing neocortex. These data further the understanding of the multifactorial RET receptor in regulating multiple signaling pathways and biological processes.

## Materials and Methods

### Cells, tissues and plasmids

Human cervical carcinoma HeLa cells and mouse embryonic NIH3T3 fibroblasts (obtained from the American Type Culture Collection), were grown in Dulbecco's modified Eagle's medium containing 10% fetal bovine serum. Embryonic brain slices were obtained from wild type CD1 pregnant mice euthanized at 14.5 days postcoitum. Cerebral cortical neurons in primary culture were prepared from CD1 mouse fetuses at E15.5 as described elsewhere [24]. Experiments were approved by the Stockholm North Ethical Committee on Animal Experiments (Permit Number: N370/09). RET mutants were harbored and expressed in PJ7Ω plasmids and subcloned into peGFP vectors (Clonetech) to make fluorescent constructs, as previously described [37].

### Reagents

Reagents and concentrations, unless otherwise stated, were as follows: GDNF (100 ng/ml, R&D Systems), GFRα1/FC chimera (400 ng/ml, R&D Systems), U73122 (5 µM, Sigma-Aldrich), U73343 (5 µM, Sigma-Aldrich), 2-aminoethoxydiphenyl borate (2-APB, 5 µM, Sigma-Aldrich), Ryanodine (20 µM, Sigma-Aldrich), Dantrolene (10 µM, Tocris), Thapsigargin (1 µM, Sigma-Aldrich), and bis(2-aminophenoxy)ethane tetraacetic acid (BAPTA, 10 µM, Molecular-Probes).

### Cytosolic Ca^2+^ imaging

Cells were loaded with the Ca^2+^-sensitive fluorescence indicator Fura-2/AM (5 µM, Molecular-Probes) in cell culture medium at 37°C with 5% CO_2_ for 30 min. The Ca^2+^ measurements were conducted at 37°C in a heat-controlled chamber (Warner Instruments) with a cooled back-illuminated EMCCD camera Cascade II:512 (Photometrics) mounted on an inverted microscope Axiovert 100M (Carl Zeiss) equipped with a LCI Plan-Neofluar 25×/0.8NA water immersion lens (Carl Zeiss). Excitation at 340, 380 and 495 nm took place using a Lambda LS xenon-arc lamp (Sutter Instrument) equipped with a Lambda 10-3 filter-wheel (Sutter Instrument) and a *Smart*Shutter (Sutter Instrument). Emission wavelengths were detected at 510 nm, and the sampling frequency was set to 0.2 to 1 Hz. MetaFluor software (Molecular Devices) was used to control all devices and to analyze the acquired images. The experiments were performed in Krebs-Ringer's buffer containing 119.0 mM NaCl, 2.5 mM KCl, 2.5 mM CaCl_2_, 1.3 mM MgCl_2_, 1.0 mM NaH_2_PO_4_, 20.0 mM Hepes (pH 7.4), and 11.0 mM dextrose. Drugs were bath-applied.

### Western blotting

Cells were lysed by sonication and protein concentrations were determined using a BCA protein assay (Pierce). Equal amounts of cellular protein were separated by sodium dodecyl sulphate gel electrophoresis, followed by wet transfer to PVDF membranes. Membranes were blocked in 5% skim milk in Tris-buffered saline solution plus 0.5% Tween-20 for 1 h before being incubated with primary antibodies (1∶1000 ERK1/2, 1∶1000 pERK1/2, 1∶1000 pCaMKII, 1∶1000 Cleaved Caspase-3, all from Cell Signaling, 1∶1000 pPLCγ, 1∶2000 RET H-300 from Santa Cruz or 1∶200 RET AF482 from R&D Systems) overnight at 4°C and re-incubated with horseradish peroxidase-conjugated secondary antibody (1∶5000–10000 from GE Healthcare) for 1 h. Immunoreactive bands were visualized using an enhanced chemiluminescence kit (GE Healthcare).

### Immunohistochemistry

Mouse brain slices were cut (30–100 µm) with a vibratome (Leica), fixed with 4% PFA overnight at 4°C and then incubated in a blocking solution (PBS, 5% Normal Goat serum, 0.1–0.3% Triton X100, 1% Bovine Serum Albumin) for 1 h at 24°C. Blocking solution was replaced by washing solution (PBS, 0.5% Normal Goat serum, 0.3% Triton X100, 1% Bovine Serum Albumin) containing the appropriate dilution of the primary antibody overnight at 4°C. Primary antibodies used were anti-RET (1/1000, R&D Systems) or anti-Tuj1 (1/400, Millipore). Alexa Fluor 488 and 555 secondary antibodies (Invitrogen) were used to reveal the primary antibodies (1/1000, 1–2 h, 24°C). TO-PRO-3 (Invitrogen) and only secondary antibody staining were used as control (Figure S3). Slices were mounted in Glycergel (Invitrogen) and observed using confocal microscopy (Carl Zeiss LSM 5 Exciter). Images were processed using the Fiji software (NIH).

### Cell motility assay

Cells transfected with RET^WT^ and RET^1015^ were seeded in plastic culture dishes and grown to confluence. A 1.2-mm wide region devoid of cells was made in the dish using a 200-µl plastic pipette tip held against a caliper. Cells were starved overnight and then pretreated with the Ca^2+^ inhibitors for 30 min before GDNF and GFRα1 stimulation for 6–8 h. Quantification of cells moving into the empty region were performed by counting the number of cells within equal areas using bright-field light microscopy. Images were captured using a digital camera (Olympus C-7070) and processed in the Photoshop Lightroom software (Adobe).

### siRNA knock down

Cells were transfected with 80 nM specific small interfering RNA (siRNA) against PLC (PLCG1 ON-TARGETplus SMARTpool, art nr L-003559-00-0005, Dharmacon) or non-targeting siRNA (Mock-siRNA) as a control (Stealth nontarget siRNA, GC medium composition) by using Lipofectamine 2000 (Invitrogen) according to the manufacturer's protocol. Efficiency was determined by Western Blot towards PLCγ.

### Transfections and whole embryo electroporation

Transfection of HeLa cells was conducted using Lipofectamine 2000 (Invitrogen) in Opti-MEM (Invitrogen) according to the manufacturer's protocol. Electroporation of mouse embryos and organotypic brain slice culture were conducted as described previously [50], [51]. Briefly, a wild type CD1 pregnant mouse was euthanized at 14.5 days postcoitum and the embryos were taken out. A glass capillary was inserted into the lateral ventricle of the embryos, and 2–4 µl of 0.5 µg/µl RET^WT^ or RET^1015^ plasmids with 0.01% Fast Green FCF (Sigma-Aldrich) in phosphate buffered saline (PBS) were injected (Figure 6A). After injection, the forebrain area of an embryo was held with a forceps-type electrode (BTX Harvard Apparatus) with the anode on the dorsal cortical side of the injected ventricle and five cycles of square electric pulses (50 V, 50 ms) with 950 ms intervals were delivered to the embryo using an electroporator (BTX Harvard Apparatus). Experiments were approved by the Stockholm North Ethical Committee on Animal Experiments (Permit Number: N370/09).

After electroporation, coronal slices (300 µm) of the forebrain were obtained using a vibratome (Leica) and cultured in Neurobasal medium supplemented with B27. A maximum of three beads (Cibacron blue CGA, Sigma-Aldrich) of diameter ∼100 µm soaked (4–6 h, 4°C) in GDNF (500 ng/ml) or in PBS were placed in the cortical plate (CP) as described [10]. After 48–72 h, slices were fixed with PFA 4% overnight at 4°C and stained with TO-PRO-3 (Invitrogen). For each separate condition, superimposed z-stack confocal images were analyzed for number of GFP-positive progenitors per µm^3^ in a spherical region with diameter twice of the GDNF-bead (Figure 6C and D). A region in a similar location of the slice, more than ∼400 µm away from the beads, was used to normalize the results. Experiments where beads were misplaced to other areas than the CP were discarded from the analysis.

### Reverse Transcription and Real Time PCR

Total RNA was extracted from cultured cortical neurons using RNeasy kit (Qiagen). RNA quality and quantity were measured with Nanodrop 2000 (Thermo Scientific). RNA was then treated with DNaseI (Biolabs) and reverse transcription was conducted with Superscript II reverse transcriptase (Invitrogen) according to manufacturer's protocol. cDNA from NIH3T3 cells and mouse E15.5 cerebellum served as negative and positive control, respectively. Reverse transcription-PCR analysis was performed using Taq Polymerase (Invitrogen), and primers were as follows: RET forward GTACACAAACACACTCCTCTCAGG, reverse CAGGCTCCTGTTGAGAATCAG, TATA-box binding protein (TBP) forward GGGGAGCTGTGATGTGAAGT, reverse CCAGGAAATAATTCTGGCTCA. The thermal cycling conditions were: 94°C for 4 min, 35 cycles of 94°C (30 s), 60°C (30 s), and 72°C (30 s) and at the end 72°C for 4 min. The PCR products were separated on 2% agarose gel and visualized under ChemiDoc XRS+ (Biorad) after staining with GelRed (Biotium). Real-time PCR was performed in triplicates using SYBR green PCR master mix according to the manufacture's instruction (Applied Biosystems) in a 7900HT Fast real-time PCR system (Applied Biosystems). Products were analyzed with ABI 7900HT Sequence Detection System (Applied Biosystems). 2^−ΔCt^ values were used to calculate the relative expression levels and were given as mean ± SEM.

### Data analysis

The Ca^2+^ recording data were normalized and cells were considered responsive to a treatment if the mean fluorescence was increased by at least 25% over the baseline. The data were presented as means ± SEM. Student's *t*-test was used and significance was accepted at *P*<0.05.

## Supporting Information

Figure S1 **Western blotting of HeLa cells transfected with RET^WT^ or RET^1015^.** (**A**) Cells expressing RET^WT^ or RET^1015^ treated with GDNF (100 ng/ml) show normal phosphorylation of RET Tyrosine residues. (**B**) Small interfering RNA (siRNA) against PLCγ (PLCγ-siRNA) knocked-down the PLCγ protein level in HeLa cells expressing RET^WT^.
(PDF)

Figure S2 **GDNF/RET-induced Ca^2+^ signalling is inhibited by BAPTA.** Representative single-cell Ca^2+^ recording of a Fura-2/AM-loaded HeLa cell transfected with RET^WT^ and treated with GDNF (100 ng/ml). Quenching intracellular Ca^2+^ with BAPTA (10 µM) abolishes the GDNF/RET-triggered Ca^2+^ response.
(PDF)

Figure S3 **Immunohistochemistry control of embryonic cortex with only secondary antibodies.** Immunohistochemistry of an E14.5 mouse forebrain cortex coronal slice (**A**, Scale bar, 250 µm) and cortical plate (CP), intermediate zone (IZ) and ventricular zone (VZ) regions (**B**, Scale bars, 25 µm) with only secondary antibodies Alexa488 and Alexa555.
(PDF)
