# Supplementary figures and images for: RET PLCγ Phosphotyrosine Binding Domain Regulates Ca2+ Signaling and Neocortical Neuronal Migration
Source: PLoS One. 2012 Feb 15;7(2):e31258. doi: 10.1371/journal.pone.0031258 (PMC3280273; doi:10.1371/journal.pone.0031258)

***Supplementary Figure S1 - Lundgren et al.***

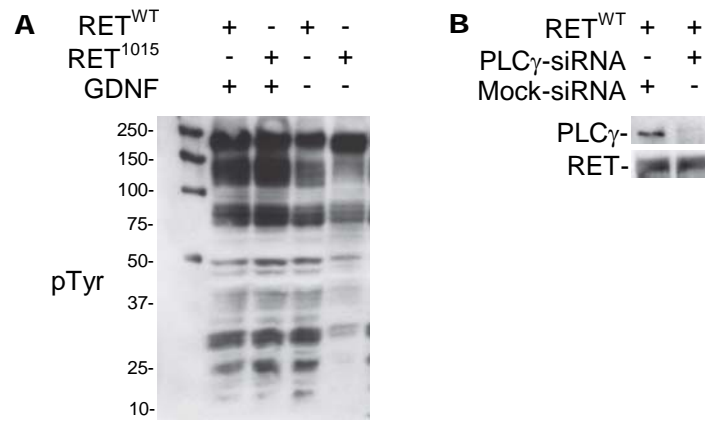

Supplement: Figure S1 — Western blotting of HeLa cells transfected with RETWT or RET1015. (A) Cells expressing RETWT or RET1015 treated with GDNF (100 ng/ml) show normal phosphorylation of RET Tyrosine residues. (B) Small interfering RNA (siRNA) against PLCγ (PLCγ-siRNA) knocked-down the PLCγ protein level in HeLa cells expressing RETWT. (PDF) [file pone.0031258.s001.pdf]

***Supplementary Figure S2 - Lundgren et al.***

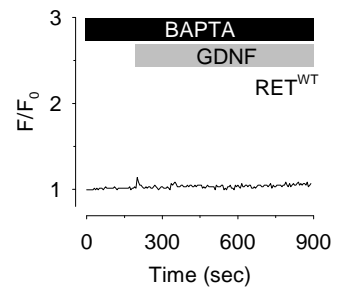

Supplement: Figure S2 — GDNF/RET-induced Ca2+ signalling is inhibited by BAPTA. Representative single-cell Ca2+ recording of a Fura-2/AM-loaded HeLa cell transfected with RETWT and treated with GDNF (100 ng/ml). Quenching intracellular Ca2+ with BAPTA (10 µM) abolishes the GDNF/RET-triggered Ca2+ response. (PDF) [file pone.0031258.s002.pdf]

***Supplementary Figure S3 - Lundgren et al.***

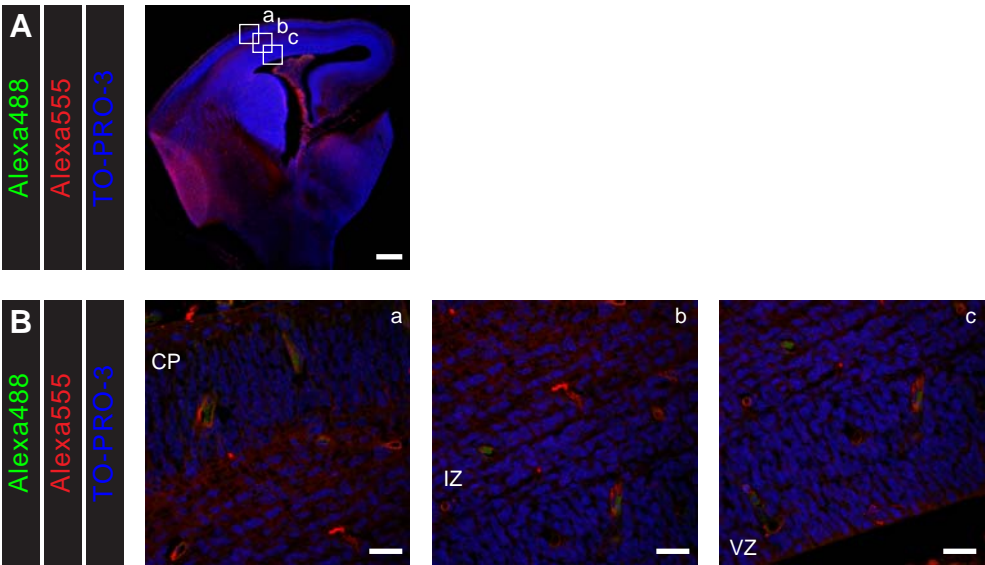

Supplement: Figure S3 — Immunohistochemistry control of embryonic cortex with only secondary antibodies. Immunohistochemistry of an E14.5 mouse forebrain cortex coronal slice (A, Scale bar, 250 µm) and cortical plate (CP), intermediate zone (IZ) and ventricular zone (VZ) regions (B, Scale bars, 25 µm) with only secondary antibodies Alexa488 and Alexa555. (PDF) [file pone.0031258.s003.pdf]
